# A Radiogenomic Approach for Decoding Molecular Mechanisms Underlying Tumor Progression in Prostate Cancer

**DOI:** 10.3390/cancers11091293

**Published:** 2019-09-02

**Authors:** Sarah Fischer, Mohamed Tahoun, Bastian Klaan, Kolja M. Thierfelder, Marc-André Weber, Bernd J. Krause, Oliver Hakenberg, Georg Fuellen, Mohamed Hamed

**Affiliations:** 1 Institute for Biostatistics and Informatics in Medicine and Ageing Research, Rostock University Medical Center, 18057 Rostock, Germany; 2 Computer Science Department, Faculty of Computers and Informatics, Suez Canal University, Ismailia 41522, Egypt; 3 Institute of Diagnostic and Interventional Radiology, Pediatric Radiology and Neuroradiology, Rostock University Medical Center, 18057 Rostock, Germany; 4 Department of Nuclear Medicine, Rostock University Medical Center, 18057 Rostock, Germany; 5 Department of Urology, Rostock University Medical Center, 18057 Rostock, Germany

**Keywords:** prostate cancer, radiogenomics, gene expression, miRNA expression, data integration

## Abstract

Prostate cancer (PCa) is a genetically heterogeneous cancer entity that causes challenges in pre-treatment clinical evaluation, such as the correct identification of the tumor stage. Conventional clinical tests based on digital rectal examination, Prostate-Specific Antigen (PSA) levels, and Gleason score still lack accuracy for stage prediction. We hypothesize that unraveling the molecular mechanisms underlying PCa staging via integrative analysis of multi-OMICs data could significantly improve the prediction accuracy for PCa pathological stages. We present a radiogenomic approach comprising clinical, imaging, and two genomic (gene and miRNA expression) datasets for 298 PCa patients. Comprehensive analysis of gene and miRNA expression profiles for two frequent PCa stages (T2c and T3b) unraveled the molecular characteristics for each stage and the corresponding gene regulatory interaction network that may drive tumor upstaging from T2c to T3b. Furthermore, four biomarkers (*ANPEP*, *mir-217*, *mir-592*, *mir-6715b*) were found to distinguish between the two PCa stages and were highly correlated (average r = ± 0.75) with corresponding aggressiveness-related imaging features in both tumor stages. When combined with related clinical features, these biomarkers markedly improved the prediction accuracy for the pathological stage. Our prediction model exhibits high potential to yield clinically relevant results for characterizing PCa aggressiveness.

## 1. Introduction

Prostate cancer (PCa) is the second most common cancer and affects millions of men every year [1,2]. Around 16% of men are diagnosed with PCa in their lifetime in the US [3]. Due to the complex and heterogenic nature of PCa, many cellular pathways and molecular mechanisms underlying PCa progression and upstaging from one stage to another have not been uncovered yet.

The prognosis and determination of best treatment strategies for PCa patients depend on the correct estimation of PCa TNM (Tumor-Node-Metastasis)-stages, based on the universal TNM tumor stage classification, which refer to the degree by which cancer has spread inside the prostate, to the nearby tissues such as seminal vesicles and bladder, and beyond [4]. Traditional classification approaches that are based on histological examination of transrectal biopsy samples and clinical parameters exhibit important shortcomings related to tumor heterogeneity, the invasive collection of tumor tissue, and the failure to distinguish between clinically relevant grades/stages of cancer [5]. For instance, serum Prostate Specific Antigen (PSA) testing is limited by its low specificity and low sensitivity for the detection of clinically significant PCa [6]. Therefore, the diagnostic power of an abnormal PSA value alone is relatively poor [7]. An additional histological examination of a set of tissue samples (biopsies) is therefore required to diagnose PCa and to assess the degree of PCa aggressiveness in the form of the Gleason score. Besides the invasiveness of biopsies, PCa also exhibits spatial heterogeneity that confounds correct assessment of tumor stage and hence often leads to understaging [8]. A digital rectal examination (DRE) is used to identify the clinical stage, which, together with PSA level and Gleason score, is used for the prediction of the pathological stage which is determined after radical surgery, but may not be predicted correctly because of under-sampling of tissue by the biopsies.

The ability to predict the pathological PCa stage clinically as precisely as possible would enable clinicians to better determine optimal treatment strategies. Furthermore, PCa patients would benefit from avoiding the potential side effects that are associated with surgical overtreatment [9,10]. Based on the PSA value, the Gleason score and the clinical stage, the Partin tables [11] provide clinicians with a prediction of the pathological stage according to the UICC TNM 8 scheme [12]. The Partin tables consider clinical stages cT1c (clinically inapparent tumor, not palpable or visible by imaging), cT2a and cT2b/cT2c (tumor confined within prostate). cT3 (tumor extends outside the prostate) and cT4 (tumor invades nearby organs) [13]. The Partin tables are an example of a nomogram, designed to predict pathological outcome based on several clinical variables [14]. Other examples of PCa nomograms are the Memorial Sloan-Kettering (MSK) nomograms (ex: the pre-radical prostatectomy) [15] and the five-point Likert scale [16], which are employed to predict the long-term results following radical prostatectomy, and to predict clinically significant prostate cancer, respectively. However, although clinically useful, the prediction accuracy of these nomograms is far from perfect [17,18,19] and therefore, PCa staging remains a critical challenge.

Recent advances in high-throughput Omics and imaging technologies and their applications in precision medicine have created new avenues for better characterizing cancer stages and for detecting molecular biomarkers for early diagnosis and prognosis [20,21]. For instance, Betroli et al. 2016 suggested a group of 29 miRNAs that could be considered as a potential panel of biomarkers in PCa prognosis and diagnosis [22]. Other studies used either gene expression signatures or individual biomarkers to predict PCa aggressiveness [23,24,25,26,27,28,29]. These studies present a prediction improvement compared to prediction based on traditional clinical parameters such as Gleason score and PSA level [30,31], but there is still room for improvement. This could be due to limitations in relying on one individual data type (gene expression or miRNA expression or other data). Also, the long follow-up time after surgery needed to observe the final endpoint such as cancer-specific mortality or PSA recurrence is hindering the assessment of prediction quality. At the same time, imaging modalities, especially multiparametric MRI (mpMRI), are used in the clinics for improving PCa diagnoses. For example, Stoyanova et al., 2016 and 2017 suggested radiogenomic models for correlating mpMRI imaging features with clinical and molecular signatures [3,32].

In this study, we performed a comprehensive integrated analysis of gene and miRNA expression profiles for PCa patients with the two frequently reported yet highly distinctive clinical stages T2c and T3b. We characterized the exclusively differentially expressed genes and miRNAs, and the associated functional terms and pathways for each stage. We further identified candidate biomarkers (*ANPEP*, *mir-217*, *mir-592*, *mir-6715b*), which markedly improved the prediction accuracy for pathological stage, when combined with conventional clinical features. Notably, these biomarkers are highly correlated with aggressiveness-related imaging features such as the tumor volume intensity and the tumor texture features. Furthermore, we unraveled the potential molecular interactions underlying PCa progression/upstaging from stage T2c to stage T3b via construction of a PCa-GRN network and highlighted four central hub miRNAs that could conceivably drive the upstaging process between these two stages. This proof of principle study demonstrates a plausible association of carcinogenic gene and miRNA expression with PCa upstaging and progression, establishes the potential of improving the prediction accuracy of pathological stages of PCa patients via integrating clinical and molecular features, and finally sheds light on the promise of integrating imaging with molecular and clinical data (radiogenomics) for clinical decision-making.

## 2. Results and Discussion

### 2.1. Description of the Radiogenomic Approach

**Figure 1:**
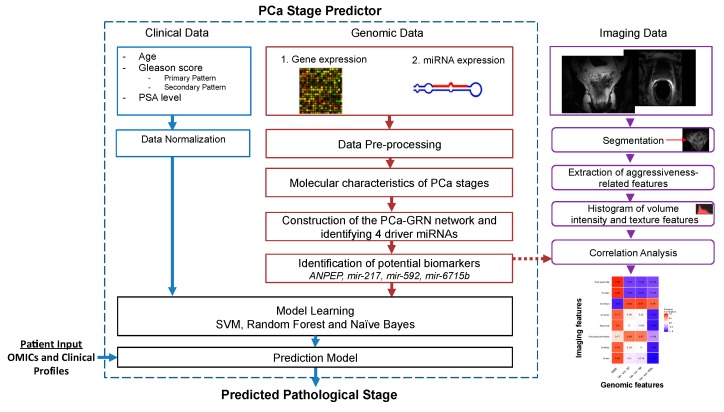
A schematic diagram for the radiogenomic approach involving clinical, genomic, and imaging datasets for prostate cancer (PCa).

We implemented and applied a radiogenomic approach aiming at unraveling the molecular mechanisms underlying PCa aggressiveness and upstaging and utilizing the clinical data together with imaging and genomic data to improve the accuracy of predicting the pathological stage. As a case study, we directed our analysis to the two frequent yet highly distinctive PCa stages T2c and T3b. We processed clinical data (age, PSA level, Gleason score) and two types of transcriptomic data (gene and miRNA expression data, from the TCGA archive) for 298 primary PCa tumor cases (T2c and T3b) and 52 healthy cases. The clinical and pathological characteristics of the PCa tumor cohort are listed in Table 1. For revealing the functional characteristics of both stages, we identified stage-specific genes and miRNAs. This was performed in two steps: first, we characterized the differentially expressed genes and miRNAs between the samples from stages T2c and T3b and the 52 healthy samples. Second, we excluded those generic PCa-related genes and miRNAs, which are differentially expressed between all the 496 available tumor samples and the 52 available healthy samples. Imaging data were available only for 14 matching cases for both stages (6 images for T2c and 8 for T3b) on the TCIA archive. Consequently, the available imaging data were not sufficient for training the prediction model. Therefore, we used it for assessing the identified molecular biomarkers via correlation analysis. Figure 1 illustrates our integrative approach for the joint analysis of clinical, genomic, and imaging data of PCa patients. It starts with the separate pre-processing of each dataset, followed by unraveling the molecular and functional characteristics (e.g., stage-specific genes and miRNAs, GO functional terms, and KEGG pathways) for each stage. Next, we constructed the gene regulatory network (GRN) that likely drives tumor upstaging from the T2c stage to the T3b stage, and consequently identifies potential gene and miRNA biomarkers from the genomic data, based on a strict differential expression criterion. The expression signatures of these biomarkers were correlated with aggressiveness-related imaging features for both stages T2c and T3b. Finally, the expression profiles of the identified biomarkers were combined with the clinical data to train classifiers and predict the pathological stage.

### 2.2. Functional Characteristics of T2c and T3b Stages

**Figure 2:**
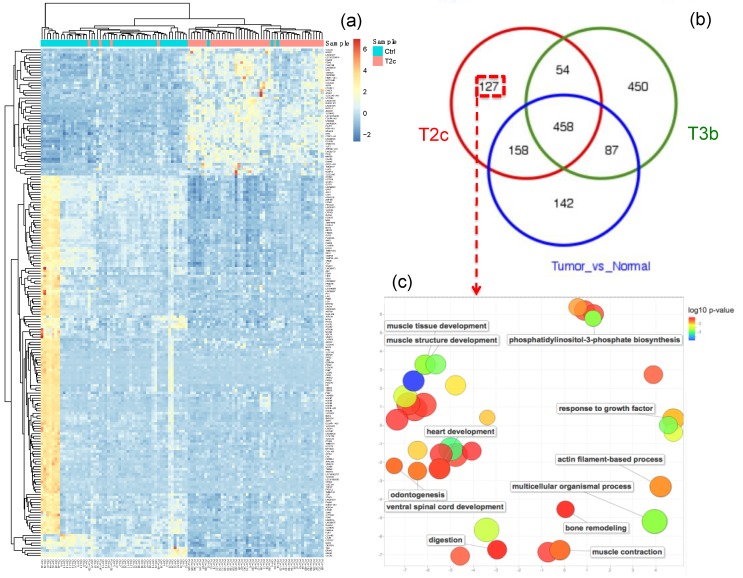
Functional characteristics of genes specific to the T2c stage in prostate cancer (PCa). (**a**) The heatmap for the top 200 differentially expressed genes (DEG) between T2c tumor samples and healthy samples. Blue denotes down-regulation whereas red-yellow denotes up-regulation. The dendrograms on the upper and left sides show the hierarchical clustering tree of samples and genes, respectively. (**b**) A Venn diagram of the overlap between the DEGs identified for: T2c stage versus healthy samples, T3b stage versus healthy samples, and all tumor samples versus healthy samples. (**c**) A scatter plot shows the visualization of the top enriched generic GO terms of the 127 genes, that are exclusively deregulated in the T2c tumor samples, based on the GO semantic similarities. GO term node colors indicate the p-values for the enrichment of the GO terms. These generic GO terms represent implicitly their subterms, which are not visualized in the plot, but listed in Supplementary Table S1. The scatter plot was generated using the web tool REVIGO [37]. The original data for Figure 2 was shown in Supplementary Materials.

**Figure 3:**
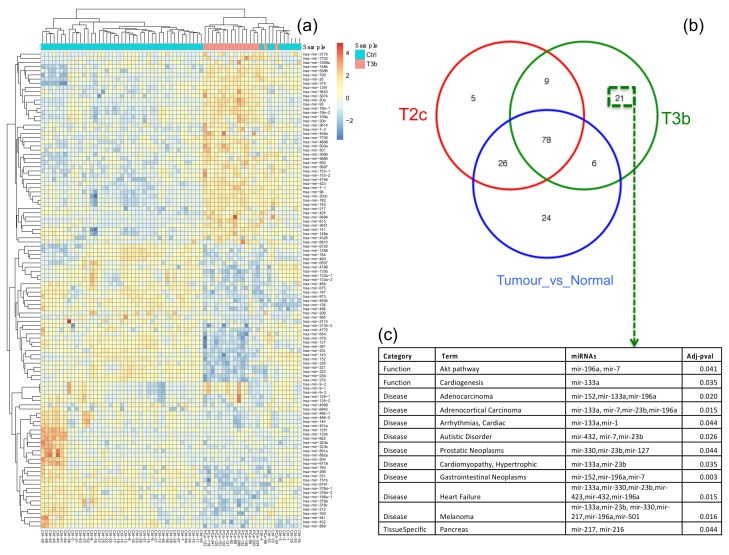
Functional characteristics of miRNAs specific to the T3b stage in prostate cancer (PCa). (**a**) The heatmap for the top 100 differentially expressed (DE) miRNAs between T3b tumor samples and healthy samples. Blue denotes down-regulation whereas red-yellow denotes up-regulation. The dendrograms on the upper and left sides show the hierarchical clustering tree of samples and miRNAs, respectively. (**b**) A Venn diagram of the overlap between the DE miRNAs identified for: T2c versus healthy samples, T3b stage versus healthy samples, and all tumor samples versus healthy samples. (**c**) A table lists the enriched functional terms, enriched diseases, and tissue specificity of the 21 miRNAs that are exclusively deregulated in the T3b tumor samples. The original data for Figure 3 was shown in Supplementary Materials.

Differential expression (DE) analysis resulted in 127 genes and 5 miRNAs that characterize (i.e., that are specifically differentially expressed in) the T2c stage. This was done by comparing the T2c samples to the healthy samples and selecting the DE genes/miRNAs which were not identified in the comparison of the T3b samples to the healthy samples, nor identified in the comparison of all tumor samples to all healthy samples, see Figure 2 (genes) and Figure S1 (miRNAs). Similarly, 450 genes and 21 miRNAs were found to be specifically DE for the T3b stage samples, see Figure 3 (miRNAs) and Figure S2 (genes). The functional characteristics of each tumor stage were backed up by inspecting the associated GO biological processes and KEGG pathways via conducting a functional enrichment analysis. The functional analyses of stage-specific genes and miRNAs revealed diverse functional GO terms and KEGG pathways for each stage. We thus listed the significant pathways that were enriched in the specific genes for stage T2c and stage T3b, which may be of relevance to the etiology of the corresponding PCa stage, in Table 2. Moreover, Figure 2c visualizes the generic GO terms enriched within the T2c-specific genes, while in Table S1 the top 20 significant GO terms and the underlying gene sets are listed. Matching with the “growth” theme featured by the GO terms, a frequently occurring gene family in these gene sets is the FGF or fibroblast growth factor gene family, which is known to be associated with prostate tumorigenesis [33], concordant with the T2c stage. For the T3b stage, specific genes were involved in a much wider spectrum of GO terms, see Table S2. For instance, the gene ACTG2, which is associated with prostatic stromal cells [34], was involved in seven significant GO terms. Similarly, the genes ETV1 and SRD5A2 were involved in five and four significant GO terms; they were found to be over-expressed in primary versus metastatic PCa, concordant with the T3b stage, and associated with an increased prostate cancer risk [35], respectively. A similar analysis was applied to the stage-specific miRNAs (21 for T3b and 5 for T2c) as shown in Figure 3 and Figure S1. Markedly more miRNAs and related PCa-pathways (such as the PI3k-Akt pathway [36] and prostatic neoplasms) are implicated in advanced PCa stages (Figure 3) than in earlier stages (Figure S1).

### 2.3. Construction of the Prostate Cancer (PCa-GRN) Network

**Figure 4:**
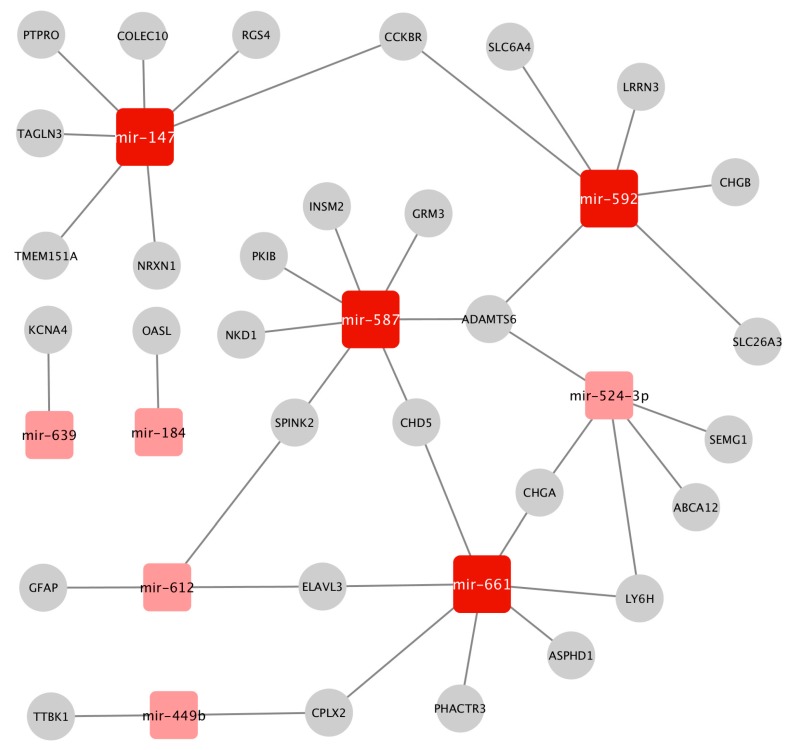
The GRN gene regulatory network prostate cancer (PCa-GRN) constructed from the miRNAs and genes differentially expressed between the T2c and the T3b tumor samples. The dark red nodes represent potential driver miRNAs. Square nodes denote the miRNAs, whereas the circular grey nodes represent genes. The network was visualized using the Cytoscape tool.

Next we performed differential expression analysis of the gene and miRNA expression data between the two stages. This yielded 125 differentially expressed (DE) genes and 19 DE miRNAs. We constructed a GRN network that combines transcriptional and post-transcriptional regulatory interactions between the DE genes and DE miRNAs (see Methods). We refer to this network as the PCa-GRN network although it represents the progression between two PCa stages only. The PCa-GRN network comprises nine miRNAs regulating 30 target genes, see Figure 4. This PCa-GRN network indicates how miRNAs are playing a critical role in the complex regulation system underlying PCa progression and upstaging as exemplified here from T2c to T3b. This is concordant with the results of the TCGA consortium analysis [1] where they found various miRNA expression patterns between different categories of PCa tumors. In order to quantify the mechanistic impact of these miRNAs and to characterize the central hub nodes that contribute essentially to the overall regulation, we computed the node degree centrality parameters and ranked the nodes according to their degrees. We identified 4 central hub miRNAs (*mir-592*, *mir-587*, *mir-147*, *mir-661*) that dominate the PCa-GRN network and maintain the interactions between these miRNAs and their neighboring genes. Hence, they could act as driver miRNAs and genetic regulators for PCa progression across the two stages. Remarkably, the aberrant expression patterns of the 4 candidate driver miRNAs have been connected to pathogenesis of various cancer types such as breast carcinoma and colorectal cancer via regulating cancer–specific pathways such as AKT/mTOR signaling pathways [38,39]. However, their molecular interactions with PCa-specific deregulated genes, and their regulation mechanisms within PCa progression were not reported before. This could highlight new insights into these candidate driver miRNAs as potential targets for new drugs delaying PCa progression.

### 2.4. Functional Homogeneity within the Prostate Cancer (PCa-GRN) Network

**Figure 5:**
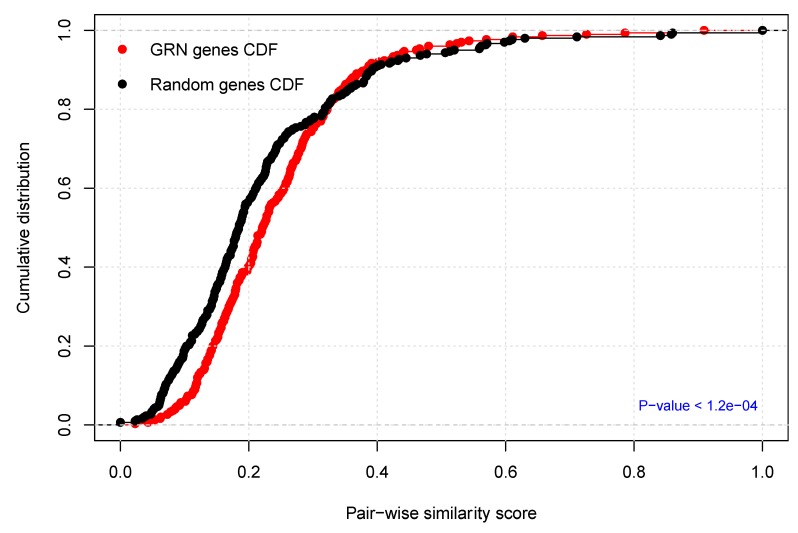
Functional homogeneity of the constructed prostate cancer (PCa-GRN) network. The plot depicts the cumulative distribution of GO functional semantic scores of gene pairs of the PCa-GRN genes (**red**) versus randomly selected genes (**black**). The p-value was calculated using the Kolmogorov–Smirnov test.

Furthermore, we evaluated the biological evidence for the PCa-GRN network in more depth to better assess the functional integrity of the biological processes underlying the etiology of PCa progression and upstaging. We estimated the functional homogeneity of the PCa-GRN genes and the target genes of the nine miRNAs by calculating the functional similarity scores between all gene pairs and comparing the resulting distribution to the similarity score distribution of randomly selected gene pairs from the PCa-GRN network. Interestingly, the PCa-GRN genes have significantly more cellular functional homogeneity than randomly selected ones (*p*-values < 1.2 × 10^−4^, Kolmogorov–Smirnov test), see Figure 5. Therefore, the nine miRNAs, and their 30 target genes comprising the PCa-GRN network could highlight new insights into a miRNA-gene based network that is representing the molecular interactions and dysregulation mechanisms underlying PCa progression from the T2c stage to the T3b stage.

### 2.5. Biomarker Identification

**Figure 6:**
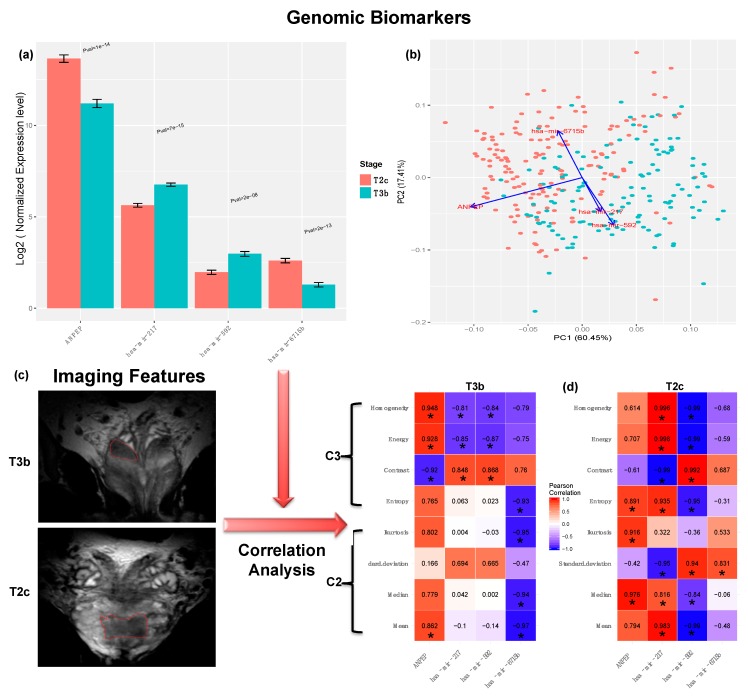
The molecular biomarkers and their correlation with the corresponding imaging features. (**a**) The normalized expression levels of the four molecular (“genomic”) biomarkers in tumor stages T2c and T3b. (**b**) Principal Component analysis (PCA) clustering of tumor samples T2c and T3b based on the normalized expression levels of the four biomarkers. (**c**) Screenshots from the Osirix software [46] for fusion of MRI delineated prostate regions of interests. We outline the prostate volume in coronal axis T2-weighted fast MRI images for both T2c and T3b samples. (**d**) The correlation matrix between the normalized expression levels of the four biomarkers and the extracted aggressiveness-related radiographic features C2 and C3. The significant correlations (*FDR* < 0.05) are marked with (*). C2 category represents the histogram of tumor volume intensity and basic statistical metrics such as mean, median, standard deviation, and kurtosis. The C3 feature category denotes the texture analysis of the tumor volume and includes Gray-Level Co-occurrence Matrix (GLCM) features such as contrast, energy, and homogeneity metrics [3]. The original data for Figure 6 was shown in Supplementary Materials.

To identify distinguishing biomarkers of genes and miRNAs between the two PCa stages, we restrained the FDR cutoff to 0.001 and increased the fold change threshold to 2.5-fold. Intriguingly, we identified the DE gene *ANPEP* and the DE miRNAs *mir-217*, *mir-592*, *mir-6715b*, that survived the above criteria. The gene *ANPEP* and the miRNA *mir-6175b* were downregulated in the T3b tumor samples while the miRNAs mir-217 and mir-592 were downregulated in the T2c tumor samples, see Figure 6a. PCA analysis of the normalized expression profiles for these four biomarkers revealed reasonable separation between the T2c and the T3b samples, see Figure 6b. Interestingly, many studies reported the aberrant expression patterns of the *ANPEP* gene in PCa cells [40,41] and furthermore *ANPEP* was suggested to be a potential prognostic biomarker for PCa patients [42]. Many studies reported the implications of *mir-217*, *mir-592*, *mir-6715b* either as a potential therapeutics to enhance chemosensitivity response in PCa [43], or in promoting proliferation of PCa cancer cells [44], or in the pathogenesis of other cancer types [45], respectively. However, their potential as molecular biomarkers in clinical outcomes has not been outlined before. Hence, miRNAs *mir-217*, *mir-592*, *and mir-6715b* are novel candidate biopsy-derived diagnostic biomarkers for PCa stages as will be demonstrated in the next section. To unravel the deregulation mechanisms of these four potential biomarkers, we investigated their association (see Methods) with other features from DNA methylation, somatic mutations, and protein expression levels of PCa samples, see Figures S3–S5. For instance, the expression of *ANPEP* was inversely proportional to the hypermethylation of many differentially methylated regions (DMRs) and CpG islands (Figure S3c). This may explain the mechanisms of *ANPEP* downregulation in advanced stages of PCa. Further research work is warranted to comprehensively analyze the implications of such observations in PCa progression and diagnosis.

### 2.6. Correlation Analysis with Aggressiveness-Related Imaging Features

To assess the biological relevance of our candidate biomarkers with respect to PCa progression and upstaging, we investigated their correlation to aggressiveness-related imaging features extracted from corresponding MRI images of PCa patients with the two stages under investigation (T2c and T3b), see Methods. We extracted the feature classes C2 and C3 which are related to PCa aggressiveness and upstaging as described by Stoyanova et al., 2016 [3]. The C2 class describes the distribution of intensities of tumor volume intensity and basic statistical metrics such as mean, median, standard deviation, and kurtosis. The C3 feature class refers to the texture analysis of the tumor volume including the Haralick GLCM features: contrast, energy, homogeneity, and entropy metrics, see Figure 6c. We computed the Pearson correlation between the expression profiles of our candidate biomarkers and the C2 and C3 imaging features for both stage groups (T2c and T3b). Figure 6d shows relatively high positive and negative correlation between the aggressiveness–related features and the expression signatures of the four biomarkers, especially *ANPEP* (max r = 0.97 and min *p*-value = 0.032) in T2c patients, and max r = 0.94 and min *p*-value = 0.025 in T3b patients. Significant correlations are marked by an asterisk. It is also noteworthy that the C3 features exhibited significant differential correlation between T2c patients T3b patients. For the T3b patient group, the C3 features (except the entropy) were found to be much better than the C2 features in correlating with the biomarker expression signatures. This also matches the findings about the plausible diagnostic power of the C3 feature class especially in advanced PCa stages [47].

### 2.7. Assessing the Predictive Power of the Identified Biomarkers

We randomly partitioned the data into two stratified sample sets, the training data (70%) and the test data (30%) and performed the biomarker identification again only on the training dataset (the 70% of the data). We repeated the aforementioned procedure for multiple runs (10 times), see Methods. In fact, the set of biomarkers did not change and we explain this invariance by the strict selection criteria (2.5 fold and FDR of 0.001) we used for identifying them, and we also note their power in separating the two classes as shown by the PCA analysis in Figure 6b. For each run, we performed the training and testing procedure, the results of which are described next.

**Figure 7:**
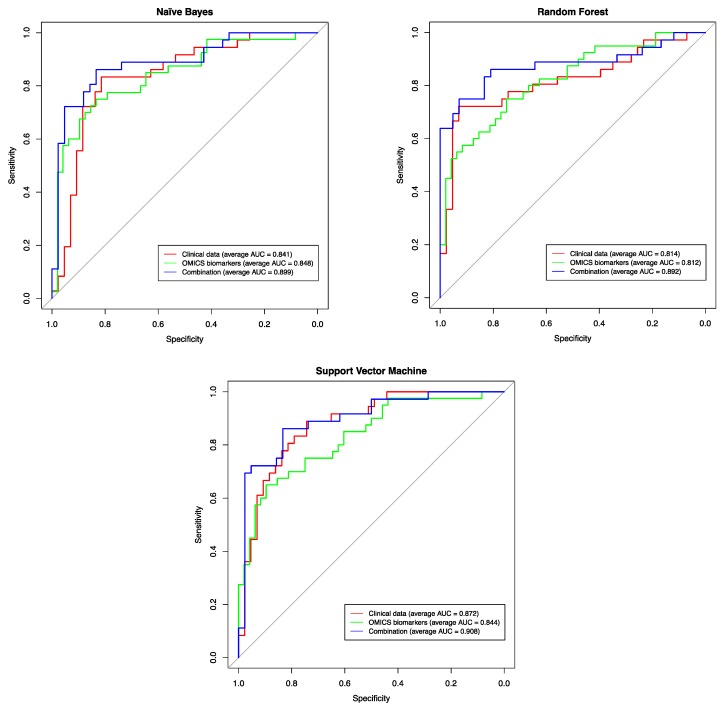
Prediction performance using the clinical features, the genomic features, and the combined feature set, for predicting the pathological stage (T2c versus T3b). The shown receiver operating characteristic (ROC) curves are representatives of the ROC curves obtained from the three prediction methods, selected because their area under the curve (AUC) value is closest to the average AUC value of the respective method over all 10 runs, see Supplementary Table S8.

In order to evaluate our candidate biomarkers as diagnostic features for predicting the corresponding pathological stages, we used three machine-learning methods (Naïve Bayes, Support Vector Machines (SVM) and Random Forest) on three feature sets: (1) the clinical features only (age, PSA level, and Gleason score), (2) the molecular features only (the expression profiles of the candidate biomarkers *ANPEP*, *mir-217*, *mir-592*, *mir-6715b*), and (3) the combined set (all 7 features). We used three machine-learning methods to ensure the reliability of the prediction efficacy of all used features. Theoretical backgrounds about the three used machine-learning methods and the parameters used for training and testing processes are described in full details in the Supplementary files. Figure 7 exemplifies the prediction accuracy by receiver operating characteristic (ROC) curves for each method on the three feature sets described above. Prediction accuracy was evaluated using the area under the ROC curve (AUC), which measures the ability of a method (based on the respective set of features) to differentiate between the pathological stages. All prediction results are tabulated in Supplementary Table S8.

The results revealed that the predictions using the molecular features only (AUC = 0.844, AUC = 0.812 and AUC = 0.848 for SVM, Random Forest and Naïve Bayes, respectively, see Supplementary Table S8) show a slightly lower or equal accuracy, when compared to the predictions using clinical features only (AUC = 0.872, AUC = 0.814, and AUC = 0.841 for SVM, Random Forest, and Naïve Bayes, respectively). In line with our findings, Shen et al. were able to differentiate PCa patients with different degrees of aggressiveness using a different set of four miRNA biomarkers (*mir-20a*, *mir-21*, *mir-221*, *and mir-145*) with an AUC prediction accuracy of 0.82 [48]. It might be also worthwhile to investigate the predictive power of other molecular features (e.g., differentially methylated regions (DMRs)) identified from other OMICs datasets, which were associated with the biomarkers we found, since these biomarkers yielded prediction accuracies close to the ones based on clinical features, in predicting pathological stage. Recent studies have adopted other models of machine-learning such as Fuzzy logic [19] and neural networks [49] for predicting PCa stages based on clinical features only and reported AUC values (0.82 and 0.7, respectively) comparable to those computed by our models using the clinical features as well.

Interestingly, predicting the pathological stages based on the combined set of both clinical and molecular features returned the largest AUC for all the three methods (AUC = ~0.9 for SVM, Random Forest and Naïve Bayes), thereby outperforming the prediction models based on either clinical or molecular feature sets separately. Hence, this highlights the potential usefulness of combining features from heterogeneous datasets to achieve better prognosis for PCa patients.

## 3. Materials and Methods

### 3.1. Datasets

RNA-Seq, miRNA-Seq, and clinical data for normal and prostate tumor samples were downloaded from the GDC data portal (https://portal.gdc.cancer.gov), namely the TCGA-PRAD project. For consistency, we only considered matching samples, which were common between the two datasets (RNA-Seq, and miRNA-Seq). Each TCGA sample refers to a tissue biopsy taken from a unique individual. This resulted in a total of 546 samples composed of 52 healthy control samples and 496 tumor samples with different tumor stages. The matching imaging traits for the examined tumor stages (T2c, T3b) were obtained from the Cancer Imaging Archive (TCIA) [50], i.e., again from the TCGA-PRAD project [51]. The datasets can be downloaded using the URLs listed in Supplementary Table S3.

### 3.2. Data Pre-Processing

#### 3.2.1. Genomic Data

The gene and miRNA expression datasets were obtained in raw read counts and were consequently normalized, corrected for library size, and log2-transformed using the Bioconductor [52] package DESeq2 v.1.12.4 [53] that is part of the statistical programming language R [54].

#### 3.2.2. Clinical Data

The clinical data were also normalized using quantile normalization [55] to remove the outliers that might affect the efficacy of the prediction process.

#### 3.2.3. Imaging Data

The MRI imaging data for the examined two stages were subjected to median filters for noise reduction and further segmented using the NIH ImageJ software [56] to determine the region of interest (ROI) delineating the tumor. Next, the imaging feature categories C2 and C3, which are related to the aggressiveness and upstaging of PCa as described by Stoyanova et al. 2016 [3], were extracted. Briefly, C2 category represents the histogram of tumor volume intensity and basic statistical metrics such as mean, median, standard deviation, and kurtosis. The C3 feature category concerns the texture analysis of the tumor volume and includes Gray-Level Co-occurrence Matrix (GLCM) features such as contrast, energy, and homogeneity metrics.

### 3.3. Differential Expression and Association Analysis

The DESeq2 Bioconductor package [53] was used for the differential expression analysis for both gene and miRNA expression data. Namely, genes/miRNAs that exhibited 1.5-fold changes and False Discovery Rate (FDR) cutoff of 0.05 were classified as differentially expressed (DE) genes/miRNAs. The potentially distinctive biomarkers were identified as those DE genes/miRNAs, which showed at least 2.5 -fold changes and FDR < 0.001. The association of the identified biomarkers with methylation, protein levels, and somatic mutation data was assessed using the Spearman correlation (cutoff threshold = 0.65) and the F-statistic measure (FDR < 0.05). FDR was controlled using the Benjamini–Hochberg [57] adjustment.

### 3.4. Construction of Prostate-Specific GRN Network (PRAD-GRN)

The molecular interactions between the differentially expressed (DE) genes and the DE miRNAs were compiled from the regulatory databases of the TFmiR webserver [58]. We considered only interactions that are supported by experimental evidence. Next, we reduced the entire network to a subnetwork whose target nodes or regulator nodes are known to be associated with prostate cancer in order to contextualize the network towards prostate cancer, generating the PCa-GRN. To this end, the human miRNA disease database (HMDD) [59] as well as DisGeNET [60,61] (a database for gene-disease association) were used as sources for prostate cancer-associated miRNAs and genes, respectively. Key driver genes/miRNAs were identified by determining the highly central nodes (hub nodes) via applying the degree centrality measure on the PCa-GRN. The PCa-GRN network was visualized with Cytoscape V3.3.0 [62].

### 3.5. Assessment of the Constructed Prostate Cancer (PCa-GRN) Network

#### 3.5.1. Functional Homogeneity within the PCa-GRN Genes (Semantic Validation)

In order to assess the biological relevance of the identified molecular interactions to PCa phenotype (here represented by tumor progression), we used the GoSemSim R package [63] to estimate semantic similarity scores according to the Gene Ontology (GO) annotations. Statistical significance was computed by comparing the similarity scores of the PCa-GRN genes to the similarity scores of randomly selected genes (using the same number of genes). We repeated the permutation procedure 100 times and adopted the Kolmogorov–Smirnov test to check whether the similarity scores of PCa-GRN gene pairs were statistically higher than the scores of randomly selected pairs.

#### 3.5.2. Enrichment Analysis of Genes and miRNAs

The functional annotation tool DAVID [64] was used to identify significantly enriched functional categories in the gene sets. Enrichment analysis of the miRNA sets was performed using TAM [65]. For this, we determined which functional categories were annotated to at least 2 genes/miRNAs and at the same time are significantly overrepresented in the gene/miRNA study set, as previously done in [66]. For both gene and miRNA enrichment analysis, Fisher’s Exact test was employed followed by the Benjamini–Hochberg [57] adjustment for controlling the FDR, with a cutoff value of 0.05.

### 3.6. Correlation Analysis

The pairwise correlations between the expression signatures of the identified biomarkers and the extracted aggressiveness-related imaging features was performed using Pearson correlation. The significance was computed using the R method cor.test followed by the Benjamini–Hochberg adjustment for controlling the false discovery rate (FDR), with a cutoff value of 0.05.

### 3.7. Prediction Models

We performed multiple runs (10×) with random data partitions (70% of the data for training and 30% for testing). The sampling of all partitions was stratified, so that the distribution of the two classes is proportional to the original distribution in the whole dataset. The molecular biomarkers were then identified by differential expression, see “Differential Expression and Association Analysis”, above, with the strict thresholds of at least 2.5-fold changes and FDR < 0.001. The molecular biomarkers and/or the clinical features already available, were then used to train three machine-learning methods, Naïve Bayes, Support-Vector Machine (SVM) and Random Forest. Model training and data partitioning were performed using the R caret package [67] with the default parameter settings for all classifiers. Besides the method’s own default parameter selection in the training step, no additional parameter tuning was performed.

This whole learning process was performed for every method after removing the NA entries from all datasets. Classifiers were trained based on three datasets; the clinical features only, the molecular features only and finally the combined set of both clinical and molecular features, resulting in nine classifiers that were trained and tested. The AUC (Area Under Curve) characteristic was used as the evaluation metric for the prediction results. We used the pROC package [68] for ROC (Receiver operating characteristic) analysis and for computing the averaging step over the runs to obtain one value as the average AUC for the nine classifiers. The learning and prediction parameters are described in sufficient details in Supplementary Tables S4–S6.

## 4. Conclusions

The current study presented an integrated regulatory analysis of gene and miRNA expression data for PCa samples to unravel molecular features, related GO functional terms, and pathways underlying PCa progression, and to identify potential biomarkers that can distinguish different PCa stages. The biomarkers we found belong to genes and miRNAs that play critical roles in PCa and other cancer types and showed high correlation with aggressiveness-related imaging features extracted from mp-MRI images. When combined with the traditional clinical features and using the power of machine learning, these biomarkers were able to improve the prediction accuracy of the corresponding PCa pathological stages.

To this end, future research work is demanded for predicting the best treatment strategy, such as chemotherapy, radiotherapy, or surgery. Other future research includes the development of similar integrative approaches such as those based on patient similarity networks that better take into consideration the molecular heterogeneity of PCa. This is actually one major limitation of our approach. Another limitation is the insufficient number of imaging samples that prevented us from combining the imaging features together with clinical and molecular features for enhancing the prediction of the PCa stages. If more imaging samples were provided for the different PCa stages, then such an analysis would have been easily possible. Further joint analysis of the associated molecular features (from DNA methylation, other non-coding RNAs, and somatic mutations) with the four identified biomarkers, as well as wet- lab experiments may enable to characterize more clinically relevant OMICs features that can potentially be used for diagnosis and prognosis of PCa. Finally, our approach can be applied to other cancer types or complex diseases with progressive stages and might be also extended for studying cellular developmental stages as well.

## Figures and Tables

**Table 1:** The clinical characteristics of the analyzed prostate cancer (PCa) cohort.

Pathological STAGE	Count	Age Median (Min–Max)	PSA-Value Median (Min–Max)	Gleason Score	Count	Clinical Stage	Count	Biochemical Recurrence	Count	Ethnicity	Count
				Primary + Secondary		Stage					
**T2c**	164	59 (41–77)	0.1 (0.01–14.69)	3 + 3	25	T1b	0	Yes	6	Black or African American	3
				3 + 4	84	T1c	84	No	131	White	58
				4 + 3	31	T2	4	Not available	27	Not available	103
				≥8	24	T2a	16				
						T2b	10				
						T2c	23				
						T3a	2				
						T3b	0				
						T4	0				
						Not available	25				
**T3b**	134	62 (46–78)	0.1 (0.01–37.36)	3 + 3	1	T1b	2	Yes	29	White	27
				3 + 4	8	T1c	23	No	94	Not available	107
				4 + 3	21	T2	6	Not available	11		
				≥8	104	T2a	14				
						T2b	12				
						T2c	16				
						T3a	14				
						T3b	16				
						T4	1				
						Not available	30				

**Table 2:** Potential KEGG pathways featuring the molecular mechanisms of the two prostate cancer (PCa) stages T2c and T3b.

**Enriched Pathways Which Characterize the T2c Stage but Not the T3b Stage**		
**Pathways**	**Involved Genes**	**Adj-Pval**
hsa05218: Melanoma	FGF6, FGF8, FGF23, FGF3	3.00 × 10^−3^
hsa04010: MAPK signaling pathway	FGF6, DUSP4, FGF8, FGF23, FGF3, PLA2G4D	3.00 × 10^−3^
hsa04810: Regulation of actin cytoskeleton	FGF6, FGF8, FGF23, MYLPF, FGF3	9.00 × 10^−3^
hsa04151: PI3K-Akt signaling pathway	FGF6, FGF8, COL6A5, FGF23, FGF3, EIF4E1B	1.00 × 10^−2^
hsa04014: Ras signaling pathway	FGF6, FGF8, FGF23, FGF3, PLA2G4D	1.1 × 10^−2^
hsa04015: Rap1 signaling pathway	FGF6, FGF8, FGF23, FGF3	4.7 × 10^−2^
**Enriched Pathways Which Characterize the T3b Stage But Not the T2c Stage**		
**Pathways**	**Involved Genes**	**Adj-Pval**
hsa04080: Neuroactive ligand-receptor interaction	GABRD, MCHR1, GABRA2, GABRA3, GABRB2, ADCYAP1R1, GRIA3, NTSR2, GHRHR, HRH3, PRLR, GALR1, HRH2, P2RX2, NPFFR1, CHRNA1, ADRA1D, GABRQ	2.23 × 10^−5^
hsa05033: Nicotine addiction	GABRD, GABRA2, GABRB2, GABRA3, GRIA3, GABRQ	9.70 × 10^−4^
hsa04972: Pancreatic secretion	KCNMA1, CD38, ATP2B4, PLA2G2A, PLA2G2C, CPA1, ATP1A2, PRKCB	2.09 × 10^−3^
hsa05143: African trypanosomiasis	IL6, HBA2, HBB, SELE, PRKCB	3.58 × 10^−3^
hsa04727: GABAergic synapse	GABRD, PLCL1, GABRA2, GABRB2, GABRA3, GABRQ, PRKCB	5.94 × 10^−3^
hsa04510: Focal adhesion	CAV3, CAV1, RASGRF1, PAK3, RAC3, ACTN2, ITGB3, FLNC, COL4A6, PRKCB, FN1	6.53 × 10^−3^
hsa04723: Retrograde endocannabinoid signaling	GABRD, GABRA2, GABRB2, GABRA3, GRIA3, GABRQ, PRKCB	1.34 × 10^−2^
hsa05144: Malaria	IL6, CXCL8, HBA2, HBB, SELE	1.46 × 10^−2^
hsa05146: Amoebiasis	GNAL, IL6, CXCL8, ACTN2, COL4A6, PRKCB, FN1	1.67 × 10^−2^
hsa04020: Calcium signaling pathway	GNAL, CD38, ATP2B4, ERBB4, HRH2, PLN, P2RX2, ADRA1D, PRKCB	2.27 × 10^−2^
hsa04970: Salivary secretion	KCNMA1, CD38, ATP2B4, ATP1A2, ADRA1D, PRKCB	2.53 × 10^−2^
hsa04270: Vascular smooth muscle contraction	KCNMA1, ACTG2, PLA2G2A, PLA2G2C, ADRA1D, KCNMB1, PRKCB	2.77 × 10^−2^
hsa05032: Morphine addiction	GABRD, GABRA2, GABRB2, GABRA3, GABRQ, PRKCB	3.14 × 10^−2^
hsa05205: Proteoglycans in cancer	CAV3, MIR10B, WNT16, CAV1, ERBB4, ITGB3, FLNC, PRKCB, FN1	4.02 × 10^−2^
hsa05412: Arrhythmogenic right ventricular cardiomyopathy (ARVC)	SGCG, DMD, ACTN2, ITGB3, CTNNA3	4.85 × 10^−2^

## Supplementary Materials

The following are available online at https://www.mdpi.com/2072-6694/11/9/1293/s1, Figure S1: Functional characteristics of the miRNAs specific to T2c stage in PCa. Figure S2: Functional characteristics of genes specific to the T3b stage in PCa. Figure S3: Association analysis of the ANPEB gene biomarker with other PCa OMICs datasets. Table S1: GO Functional Enrichment of the 127 genes, which are exclusively dysregulated in the T2c stage and not in the T3b stage. The table lists the top 20 significant GO Biological process (BP) terms. Table S2: GO Functional Enrichment of the 450 genes, which are exclusively dysregulated in the T3b stage and not in the T2c stage. The table lists the top 20 significant GO Biological process (BP) terms. Table S3: the download URLs of the analyzed data sets in this study. Table S4: Parameters of the random forest models constructed by the caret train method for the three different input datasets. Table S5: Parameters of naive bayes models constructed by the caret train method for the three different input datasets. Table S6: Parameters of SVM models constructed by the caret train method for the three different input datasets. Table S7: Overview of the three datasets used to train the prediction model. Table S8: the AUC performance of the different methods and datasets splitted into the 10 runs.

## References

[1] Abeshouse A., Ahn J., Akbani R., Ally A., Amin S., Andry C.D., Annala M., Aprikian A., Armenia J., Arora A. (2015). The molecular taxonomy of primary prostate cancer. Cell.

[2] Ferlay J., Soerjomataram I., Dikshit R., Eser S., Mathers C., Rebelo M., Parkin D.M., Forman D., Bray F. (2015). Cancer incidence and mortality worldwide: Sources, methods and major patterns in GLOBOCAN 2012. Int. J. Cancer.

[3] Stoyanova R., Takhar M., Tschudi Y., Ford J.C., Solórzano G., Erho N., Balagurunathan Y., Punnen S., Davicioni E., Gillies R.J. (2016). Prostate cancer radiomics and the promise of radiogenomics. Transl. Cancer Res..

[4] Walz J., Joniau S., Chun F.K., Isbarn H., Jeldres C., Yossepowitch O., Chao-Yu H., Klein E.A., Scardino P.T., Reuther A. (2011). Pathological results and rates of treatment failure in high-risk prostate cancer patients after radical prostatectomy. BJU Int..

[5] Incoronato M., Aiello M., Infante T., Cavaliere C., Grimaldi A.M., Mirabelli P., Monti S., Salvatore M. (2017). Radiogenomic analysis of oncological data: A technical survey. Int. J. Mol. Sci..

[6] Barbieri C.E., Demichelis F., Rubin M.A. (2012). Molecular genetics of prostate cancer: Emerging appreciation of genetic complexity. Histopathology.

[7] Jansen F.H., van Schaik R.H., Kurstjens J., Horninger W., Klocker H., Bektic J., Wildhagen M.F., Roobol M.J., Bangma C.H., Bartsch G. (2010). Prostate-specific antigen (PSA) isoform p2PSA in combination with total PSA and free PSA improves diagnostic accuracy in prostate cancer detection. Eur. Urol..

[8] Cookson M.S., Fleshner N.E., Soloway S.M., Fair W.R. (1997). Correlation between Gleason score of needle biopsy and radical prostatectomy specimen: Accuracy and clinical implications. J. Urol..

[9] Epstein J.I., Walsh P.C., Carmichael M., Brendler C.B. (1994). Pathologic and clinical findings to predict tumor extent of nonpalpable (stage t1 c) prostate cancer. JAMA.

[10] Epstein J.I., Pizov G., Walsh P.C. (1993). Correlation of pathologic findings with progression after radical retropubic prostatectomy. Cancer.

[11] Tosoian J.J., Chappidi M., Feng Z., Humphreys E.B., Han M., Pavlovich C.P., Epstein J.I., Partin A.W., Trock B.J. (2017). Prediction of pathological stage based on clinical stage, serum prostate-specific antigen, and biopsy Gleason score: Partin Tables in the contemporary era. BJU Int..

[12] Gospodarowicz M.K., Brierley J.D., Wittekind C. (2017). TNM Classification of Malignant Tumours.

[13] Mohler J.L., Armstrong A.J., Bahnson R.R., D’Amico A.V., Davis B.J., Eastham J.A., Enke C.A., Farrington T.A., Higano C.S., Horwitz E.M. (2016). Prostate cancer, version 1.2016. J. Natl. Compr. Cancer Netw..

[14] Dotan Z.A., Ramon J. (2009). Nomograms as a tool in predicting prostate cancer prognosis. Eur. Urol. Suppl..

[15] MSK Memorial Sloan Kettering Cancer Center. https://www.mskcc.org/nomograms/prostate.

[16] Harada T., Abe T., Kato F., Matsumoto R., Fujita H., Murai S., Miyajima N., Tsuchiya K., Maruyama S., Kudo K. (2015). Five-point Likert scaling on MRI predicts clinically significant prostate carcinoma. BMC Urol..

[17] Briganti A., Karakiewicz P.I., Joniau S., Van Poppel H. (2009). The motion: Nomograms should become a routine tool in determining prostate cancer prognosis. Eur. Urol..

[18] Chun F.K.H., Karakiewicz P.I., Briganti A., Walz J., Kattan M.W., Huland H., Graefen M. (2007). A critical appraisal of logistic regression-based nomograms, artificial neural networks, classification and regression-tree models, look-up tables and risk-group stratification models for prostate cancer. BJU Int..

[19] Cosma G., Acampora G., Brown D., Rees R.C., Khan M., Pockley A.G. (2016). Prediction of pathological stage in patients with prostate cancer: A neuro-fuzzy model. PLoS ONE.

[20] Hariri A.R., Weinberger D.R. (2003). Imaging genomics. Br. Med. Bull..

[21] Bai H.X., Lee A.M., Yang L., Zhang P., Davatzikos C., Maris J.M., Diskin S.J. (2016). Imaging genomics in cancer research: Limitations and promises. Br. J. Radiol..

[22] Bertoli G., Cava C., Castiglioni I. (2016). MicroRNAs as biomarkers for diagnosis, prognosis and theranostics in prostate cancer. Int. J. Mol. Sci..

[23] Bibikova M., Chudin E., Arsanjani A., Zhou L., Garcia E.W., Modder J., Kostelec M., Barker D., Downs T., Fan J.B. (2007). Expression signatures that correlated with Gleason score and relapse in prostate cancer. Genomics.

[24] Agell L., Hernández S., Nonell L., Lorenzo M., Puigdecanet E., de Muga S., Juanpere N., Bermudo R., Fernández P.L., Lorente J.A. (2012). A 12-gene expression signature is associated with aggressive histological in prostate cancer: SEC14L1 and TCEB1 genes are potential markers of progression. Am. J. Pathol..

[25] Bismar T.A., Demichelis F., Riva A., Kim R., Varambally S., He L., Kutok J., Aster J.C., Tang J., Kuefer R. (2006). Defining aggressive prostate cancer using a 12-gene model. Neoplasia.

[26] Cheville J.C., Karnes R.J., Therneau T.M., Kosari F., Munz J.M., Tillmans L., Basal E., Rangel L.J., Bergstralh E., Kovtun I.V. (2008). Gene panel model predictive of outcome in men at high-risk of systemic progression and death from prostate cancer after radical retropubic prostatectomy. J. Clin. Oncol..

[27] Cuzick J., Swanson G.P., Fisher G., Brothman A.R., Berney D.M., Reid J.E., Mesher D., Speights V., Stankiewicz E., Foster C.S. (2011). Prognostic value of an RNA expression signature derived from cell cycle proliferation genes in patients with prostate cancer: A retrospective study. Lancet Oncol..

[28] Larkin S., Holmes S., Cree I., Walker T., Basketter V., Bickers B., Harris S., Garbis S.D., Townsend P., Aukim-Hastie C. (2012). Identification of markers of prostate cancer progression using candidate gene expression. Br. J. Cancer.

[29] Singh D., Febbo P.G., Ross K., Jackson D.G., Manola J., Ladd C., Tamayo P., Renshaw A.A., D’Amico A.V., Richie J.P. (2002). Gene expression correlates of clinical prostate cancer behavior. Cancer Cell.

[30] Erho N., Crisan A., Vergara I.A., Mitra A.P., Ghadessi M., Buerki C., Bergstralh E.J., Kollmeyer T., Fink S., Haddad Z. (2013). Discovery and validation of a prostate cancer genomic classifier that predicts early metastasis following radical prostatectomy. PLoS ONE.

[31] Al-Kafaji G., Said H.M., Alam M.A., Al Naieb Z.T. (2018). Blood-based microRNAs as diagnostic biomarkers to discriminate localized prostate cancer from benign prostatic hyperplasia and allow cancer-risk stratification. Oncol. Lett..

[32] Stoyanova R., Pollack A., Takhar M., Lynne C., Parra N., Lam L.L., Alshalalfa M., Buerki C., Castillo R., Jorda M. (2016). Association of multiparametric MRI quantitative imaging features with prostate cancer gene expression in MRI-targeted prostate biopsies. Oncotarget.

[33] Corn P.G., Wang F., McKeehen W., Navone N. (2013). Targeting fibroblast growth factor pathways in prostate cancer. Clin. Cancer Res..

[34] Chandran U.R., Ma C., Dhir R., Bisceglia M., Lyons-Weiler M., Liang W., Michalopoulos G., Becich M., Monzon F.A. (2007). Gene expression profiles of prostate cancer reveal involvement of multiple molecular pathways in the metastatic process. BMC Cancer.

[35] Mazaris E., Tsiotras A. (2013). Molecular pathways in prostate cancer. Nephro Urol. Mon..

[36] Vivanco I., Sawyers C.L. (2002). The phosphatidylinositol 3-kinase–AKT pathway in human cancer. Nat. Rev. Cancer.

[37] Supek F., Bošnjak M., Škunca N., Šmuc T. (2011). REVIGO summarizes and visualizes long lists of gene ontology terms. PLoS ONE.

[38] Zhang Y., Zhang H.E., Liu Z. (2016). MicroRNA-147 suppresses proliferation, invasion and migration through the AKT/mTOR signaling pathway in breast cancer. Oncol. Lett..

[39] Zhang Y., Talmon G., Wang J. (2016). MicroRNA-587 antagonizes 5-FU-induced apoptosis and confers drug resistance by regulating PPP2R1B expression in colorectal cancer. Cell Death Dis..

[40] Liu A.Y., Roudier M.P., True L.D. (2004). Heterogeneity in primary and metastatic prostate cancer as defined by cell surface CD profile. Am. J. Pathol..

[41] Dall’Era M.A., True L.D., Siegel A.F., Porter M.P., Sherertz T.M., Liu A.Y. (2007). Differential expression of CD10 in prostate cancer and its clinical implication. BMC Urol..

[42] Sørensen K.D., Abildgaard M.O., Haldrup C., Ulhøi B.P., Kristensen H., Strand S., Parker C., Høyer S., Borre M., Ørntoft T.F. (2013). Prognostic significance of aberrantly silenced ANPEP expression in prostate cancer. Br. J. Cancer.

[43] Lin H.M., Nikolic I., Yang J., Castillo L., Deng N., Chan C.L., Yeung N.K., Dodson E., Elsworth B., Spielman C. (2018). MicroRNAs as potential therapeutics to enhance chemosensitivity in advanced prostate cancer. Sci. Rep..

[44] Lv Z., Rao P., Li W. (2015). MiR-592 represses FOXO3 expression and promotes the proliferation of prostate cancer cells. Int. J. Clin. Exp. Med..

[45] Giulietti M., Occhipinti G., Principato G., Piva F. (2017). Identification of candidate miRNA biomarkers for pancreatic ductal adenocarcinoma by weighted gene co-expression network analysis. Cell. Oncol. (Dordr.).

[46] Rosset A., Spadola L., Ratib O. (2004). OsiriX: An open-source software for navigating in multidimensional DICOM images. J. Digit. Imaging.

[47] Wibmer A., Hricak H., Gondo T., Matsumoto K., Veeraraghavan H., Fehr D., Zheng J., Goldman D., Moskowitz C., Fine S.W. (2015). Haralick texture analysis of prostate MRI: Utility for differentiating non-cancerous prostate from prostate cancer and differentiating prostate cancers with different Gleason scores. Eur. Radiol..

[48] Shen J., Hruby G.W., McKiernan J.M., Gurvich I., Lipsky M.J., Benson M.C., Santella R.M. (2012). Dysregulation of circulating microRNAs and prediction of aggressive prostate cancer. Prostate.

[49] Tsao C.W., Liu C.Y., Cha T.L., Wu S.T., Sun G.H., Yu D.S., Chen H.I., Chang S.Y., Chen S.C., Hsu C.Y. (2014). Artificial neural network for predicting pathological stage of clinically localized prostate cancer in a Taiwanese population. J. Chin. Med. Assoc..

[50] Clark K., Vendt B., Smith K., Freymann J., Kirby J., Koppel P., Moore S., Phillips S., Maffitt D., Pringle M. (2013). The Cancer Imaging Archive (TCIA): Maintaining and operating a public information repository. J. Digit. Imaging.

[51] The Cancer Genome Atlas Prostate Adenocarcinoma (TCGA-PRAD) Data Collection. https://portal.gdc.cancer.gov/projects/TCGA-PRAD.

[52] Gentleman R.C., Carey V.J., Bates D.M., Bolstad B., Dettling M., Dudoit S., Ellis B., Gautier L., Ge Y., Gentry J. (2004). Bioconductor: Open software development for computational biology and bioinformatics. Genome Biol..

[53] Anders S., Huber W. (2010). Differential expression analysis for sequence count data. Genome Biol..

[54] Ihaka R., Gentleman R. (1996). R: A language for data analysis and graphics. J. Comput. Graph. Stat..

[55] Gallón S., Loubes J.M., Maza E. (2013). Statistical properties of the quantile normalization method for density curve alignment. Math. Biosci..

[56] Schneider C.A., Rasband W.S., Eliceiri K.W. (2012). NIH Image to ImageJ: 25 years of image analysis. Nat. Methods.

[57] Hochberg Y., Benjamini Y. (1990). More powerful procedures for multiple significance testing. Stat. Med..

[58] Hamed M., Spaniol C., Nazarieh M., Helms V. (2015). TFmiR: A web server for constructing and analyzing disease-specific transcription factor and miRNA co-regulatory networks. Nucleic Acids Res..

[59] Li Y., Qiu C., Tu J., Geng B., Yang J., Jiang T., Cui Q. (2013). HMDD v2. 0: A database for experimentally supported human microRNA and disease associations. Nucleic Acids Res..

[60] Piñero J., Queralt-Rosinach N., Bravo À., Deu-Pons J., Bauer-Mehren A., Baron M., Sanz F., Furlong L.I. (2015). DisGeNET: A discovery platform for the dynamical exploration of human diseases and their genes. Database.

[61] Piñero J., Bravo À., Queralt-Rosinach N., Gutiérrez-Sacristán A., Deu-Pons J., Centeno E., García-García J., Sanz F., Furlong L.I. (2016). DisGeNET: A comprehensive platform integrating information on human disease-associated genes and variants. Nucleic Acids Res..

[62] Shannon P., Markiel A., Ozier O., Baliga N.S., Wang J.T., Ramage D., Amin N., Schwikowski B., Ideker T. (2003). Cytoscape: A software environment for integrated models of biomolecular interaction networks. Genome Res..

[63] Yu G., Li F., Qin Y., Bo X., Wu Y., Wang S. (2010). GOSemSim: An R package for measuring semantic similarity among GO terms and gene products. Bioinformatics.

[64] Huang D.W., Sherman B.T., Lempicki R.A. (2008). Systematic and integrative analysis of large gene lists using DAVID bioinformatics resources. Nat. Protoc..

[65] Lu M., Shi B., Wang J., Cao Q., Cui Q. (2010). TAM: A method for enrichment and depletion analysis of a microRNA category in a list of microRNAs. BMC Bioinform..

[66] Hamed M., Trumm J., Spaniol C., Sethi R., Irhimeh M.R., Fuellen G., Paulsen M., Helms V. (2017). Linking Hematopoietic Differentiation to Co-Expressed Sets of Pluripotency-Associated and Imprinted Genes and to Regulatory microRNA-Transcription Factor Motifs. PLoS ONE.

[67] Kuhn M. (2015). Caret: Classification and Regression Training. https://rdrr.io/rforge/caret/.

[68] Robin X., Turck N., Hainard A., Tiberti N., Lisacek F., Sanchez J.C., Müller M. (2011). pROC: An open-source package for R and S+ to analyze and compare ROC curves. BMC Bioinform..

